# Comparative evaluation of single-incision laparoscopic surgery versus conventional laparoscopic surgery

**DOI:** 10.6026/973206300223154

**Published:** 2026-05-31

**Authors:** Utkarsh Rajput, Mohammed Fazal, Pradeep Rathore

**Affiliations:** 1 Department of General Surgery, Government Medical College, Datia, Madhya Pradesh, India; 2 Department of General Surgery, Nagda Medical College, Sheopur, Madhya Pradesh, India

**Keywords:** Single-incision laparoscopic surgery (SILS), conventional laparoscopic surgery (CLS),, minimally invasive surgery,, post-operative pain, cosmetic outcome

## Abstract

The comparative effectiveness of single-incision laparoscopic surgery versus conventional laparoscopy remains uncertain due to concerns regarding technical complexity and outcomes. Therefore, it is of interest to compare pre-operative and post-operative outcomes between conventional laparoscopic surgery and single-incision laparoscopic surgery in 200 patients. Patients were equally divided into two groups, with evaluation of operative time, complications, post-operative pain, hospital stay, cosmetic satisfaction and overall outcomes. Single-incision laparoscopic surgery demonstrated comparable clinical outcomes with improved cosmetic satisfaction, through it involved greater technical demands. Thus, we support single-incision laparoscopic surgery as a safe and effective alternative that enhances cosmetic outcomes without compromising clinical performance.

## Background:

In the last 30 years, minimally invasive surgery has changed the face of general surgery forever. Surgical treatment of several abdominal disorders has progressed substantially since the advent of laparoscopic procedures, with better patient outcomes and less morbidity as a result compared to conventional open surgery. Conventional laparoscopic surgery (CLS) typically involves the use of three to four small incisions for the placement of trocars, allowing visualization and manipulation of surgical instruments within the abdominal cavity. When compared to open surgical treatments, this method has various benefits, such as less post-operative discomfort, quicker recovery, shorter hospital stays, less wound problems and better cosmetic results [1. 2]. Traditional laparoscopy has its benefits, but there have been continuous attempts to lessen surgical stress and enhance esthetic results. Among these developments is SLIS, an abbreviation for single-incision laparoscopic surgery, which is also known as Laparo-endoscopic single-site surgery. With SILS, a specialized multiport device or many trocars are inserted via a single incision, often made at the umbilicus, allowing the full surgical process to be performed [3]. When compared to traditional multiport laparoscopic surgery, the cosmetic benefits of the umbilical incision are clear: the scar is better concealed inside the navel's natural folds [4]. Single-incision laparoscopic surgery was first introduced in the early 1990s and has since gained increasing popularity in various surgical procedures including appendectomy, cholecystectomy, colorectal surgery and bariatric surgery. The main theoretical advantages of SILS include decreased post-operative pain, reduced risk of port-site complications, faster recovery and improved cosmetic satisfaction among patients [5]. Additionally, the single-incision technique may potentially reduce the risk of bleeding, infection and hernia formation at multiple trocar sites [6]. However, despite its potential benefits, SILS presents certain technical challenges when compared with conventional laparoscopic surgery. Because all surgical instruments are introduced through a single incision, surgeons often encounter difficulties such as instrument crowding, limited triangulation, reduced range of motion and compromised ergonomics. These technical limitations may lead to longer operative times and may require advanced laparoscopic skills and specialized instruments [7].

Time to operation, discomfort thereafter, complications and overall patient satisfaction are some of the metrics that have been used to compare SILS with traditional laparoscopic surgery. Compared to traditional laparoscopy, SILS may deliver superior cosmetic outcomes and less post-operative discomfort, according to some research. However, other studies have demonstrated that the operative time in SILS may be longer due to technical complexity and the learning curve associated with the procedure [8, 9]. Cosmetic outcome has become an increasingly important factor in surgical decision-making, especially among younger patients. The umbilical incision used in SILS often results in an almost invisible scar after healing, making it particularly appealing to patients concerned about post-operative aesthetics. Studies evaluating patient satisfaction have shown that individuals undergoing SILS frequently report higher cosmetic satisfaction compared with those undergoing conventional laparoscopic surgery [10]. Post-operative pain is another important factor when evaluating minimally invasive surgical techniques. Reduced post-operative pain can facilitate early mobilization, decrease the need for analgesics and improve overall patient recovery. Some investigators have suggested that the reduction in the number of incisions in SILS may lead to decreased post-operative pain compared with conventional laparoscopy. However, the evidence remains inconsistent and further studies are required to determine whether SILS truly offers a significant advantage in this regard [11, 12]. Furthermore, the safety profile of SILS remains an important consideration. Although many studies have demonstrated comparable complication rates between SILS and conventional laparoscopy, concerns remain regarding potential risks such as port-site hernia, wound infection and intraoperative complications. Therefore, careful evaluation of clinical outcomes is necessary before adopting SILS as a routine surgical approach [13]. Therefore, it is of interest to evaluate conventional laparoscopic surgery with single-incision laparoscopic surgery in a 200-patient sample with respect to operational outcomes, post-operative pain and duration of hospital stay, complications and cosmetic satisfaction.

## Materials and Methodology:

## Study design:

The researchers set out to compare and contrast two kinds of laparoscopic surgery traditional laparoscopic surgery (CLS) and single-incision laparoscopic surgery (SILS) in order to find out which one had better clinical outcomes for elective abdominal operations. 

## Study setting:

A tertiary care teaching hospital's Department of General Surgery conducted the research over the course of 24 months. Skilled laparoscopic surgeons who adhered to established guidelines carried out each operation.

## Study population:

The study's inclusion criteria were that patients had to have elective laparoscopic abdominal surgery in order to be eligible for participation. All participants were asked to sign an informed consent form before they could be enrolled.

## Sample size:

A total of 200 patients were included in the study and were divided into two groups:

[1] Group A: Patients undergoing Single-Incision Laparoscopic Surgery (SILS) - 100 patients

[2] Group B: Patients undergoing Conventional Laparoscopic Surgery (CLS) - 100 patients

Patients were allocated to either group based on the surgical technique used.

## Inclusion criteria:

[1] Patients aged 18-65 years.

[2] Patients undergoing elective laparoscopic abdominal surgery such as cholecystectomy or appendectomy.

[3] Patients who were fit for laparoscopic surgery under general anesthesia.

[4] Patients who provided written informed consent for participation in the study. 

## Exclusion criteria:

[1] Patients with previous major abdominal surgery.

[2] Patients with severe cardiopulmonary disease contraindicating laparoscopy.

[3] Patients requiring emergency surgery.

[4] Patients with coagulation disorders or severe systemic illness.

[5] Patients who refused to participate in the study. 

## Surgical technique:

## Single-Incision laparoscopic surgery (Group A):

The SILS group underwent a single umbilicus incision of around 2-3 cm. Several trocars or a specific single-port device were placed via this incision. The same incision was used to insert a laparoscope and other working devices. This single access site was used to perform the surgical surgery utilizing typical laparoscopic procedures. After the treatment was finished, the umbilical incision was closed with absorbable sutures, so there is hardly any visible scarring.

## Conventional laparoscopic surgery (Group B):

Three or four individual trocar ports were used in the group who had traditional laparoscopic procedures. The standard procedure included inserting a 10-mm laparoscope port at the umbilicus and many 5-mm working ports into the belly for the purpose of facilitating the placement of surgical tools. The procedure was performed following standard laparoscopic surgical techniques.

## Parameters evaluated:

The following intraoperative and post-operative parameters were assessed in both groups:

[1] Operative time (measured in minutes from incision to closure).

[2] Intraoperative complications such as bleeding or organ injury.

[3] Post-operative pain assessed using the Visual Analogue Scale (VAS) at specified post-operative intervals.

[4] Length of hospital stay (measured in days).

[5] Post-operative complications, including wound infection, bleeding, or port-site hernia.

[6] Cosmetic outcome assessed using patient satisfaction scores at follow-up.

## Post-operative follow-up:

All patients were monitored during the post-operative period and were followed up for 30 days after surgery to assess recovery, complications and patient satisfaction with cosmetic outcomes.

## Data collection:

A systematic data collection proforma was used to capture all clinical and surgical data. Patient demographics, surgical results, pain ratings after surgery, complications and length of hospital stay were among the data gathered.

## Statistical analysis:

The collected data were entered into Microsoft Excel and analyzed using Statistical Package for Social Sciences (SPSS) software version 25.0.

The following statistical methods were applied:

[1] Descriptive statistics (mean standard deviation, frequency and percentage).

[2] Independent t-test for comparison of continuous variables such as operative time and hospital stay.

[3] Chi-square test for comparison of categorical variables such as complications and patient satisfaction.

A p-value < 0.05 was considered statistically significant.

## Results:

The research compared the results of Single-Incision Laparoscopic Surgery (SILS) with those of Conventional Laparoscopic Surgery (CLS) using data from 200 patients. There were two groups made up of the patients: Group A: Single-Incision Laparoscopic Surgery - 100 patients, Group B: Conventional Laparoscopic Surgery - 100 patients. Time spent operating, pain levels after surgery, length of hospital stay and complications were among the many intraoperative and post-operative variables assessed. Patients' demographics were similar in the two groups. There was no statistically significant difference (p = 0.68) between the groups in terms of patient age; both groups had traditional laparoscopic surgery with an average age of 42.3 years, while the SILS group had an average age of 41.6 years. Both the SILS and traditional laparoscopic groups had a majority of male patients (56% and 60%, respectively). Of the total patients, 44% were female and 40% were male. Respectively, the mean BMI values were also comparable between groups (p = 0.49), indicating that both groups were demographically similar and suitable for comparison (Table 1). In the traditional laparoscopic surgery group, the mean operational time was 58.7 minutes, whereas in the SILS group it was 72.4 minutes, a significant difference (p < 0.001). The technical difficulties of operating via a single incision may explain this variation. On the other hand, there was a statistically significant difference between the two groups in terms of the average length of hospital stays: 2.6 days for the SILS group and 3.1 days for the standard laparoscopic group (p = 0.002). The SILS group had 6% of patients have intraoperative problems, while the CLS group had 5%. The difference between the two groups was not statistically significant (p = 0.76), suggesting that the safety profiles of the two methods are similar (Table 2). Post-operative pain assessment showed significantly lower pain scores in the SILS group, with a mean VAS score of 3.1, compared to 4.4 in the conventional laparoscopic group (p < 0.001), indicating better post-operative comfort in patients undergoing SILS. The SILS group had a 3% wound infection rate, whereas the CLS group had a 6% rate; nonetheless, there was no statistically significant difference between the two groups (p = 0.31). There was also no statistically significant difference (p = 0.56) in the proportion of patients who developed a port-site hernia in the SILS group and the CLS group. Cosmetic satisfaction was significantly higher in the SILS group, where 92% of patients reported high satisfaction with scar appearance, compared with 78% in the conventional laparoscopic group (p = 0.006). This difference highlights the cosmetic advantage of the single-incision technique (Table 3).

## Discussion:

As a consequence of minimally invasive surgery's significant influence on surgical practice, laparoscopic surgery has replaced open abdominal surgery as the standard method for many procedures because of its shorter recovery time, less post-operative pain and better esthetic outcomes. By performing the procedure via a single umbilical incision, a novel minimally invasive technique known as Single-Incision Laparoscopic Surgery (SILS) has been created to further decrease surgical stress. Clinical outcomes of SILS and conventional laparoscopic surgery (CLS) were compared in this study, which included 200 patients (100 in each group). Patients' demographics were similar in the two groups who participated in this research. There was no statistically significant difference (p > 0.05) between the two groups with respect to mean age; both groups were 41.6 years old. In both the SILS and CLS groups, male patients made up 56% and 60% of the total, while female patients made up 44% and 40% of the groups, respectively. Wang *et al.* also reported comparable demographics; they compared traditional laparoscopic surgery with single-incision laparoscopic surgery in a randomized study and found no significant changes in baseline characteristics [1]. To guarantee that variations in results are associated with the surgical method and not patient attributes, it is essential to establish similar demographic profiles. There was a statistically significant difference (p < 0.001) in the mean operating time between the SILS group (72.4 minutes) and the CLS group (58.7 minutes), according to the current research. Consistent with other research, this data confirms that technical difficulties such instrument crowding, poor triangulation and restricted mobility cause SILS procedures to take longer throughout the operation. The learning curve for the SILS procedure is largely to blame for the much longer operating time in the SILS group compared to traditional laparoscopic cholecystectomy, according to a meta-analysis [13].

Similarly, Cao *et al.* reported that operative time was approximately 10-20% longer in SILS procedures compared with conventional laparoscopy in gastric surgery [3]. The longer operative duration observed in the present study may also reflect the increased technical complexity associated with single-incision access. Reducing post-operative discomfort is one of the primary objectives of minimally invasive surgery. Approximately 29.5% lower pain levels were recorded in the SILS group compared to the CLS group at 24 hours (p < 0.001); with an average post-operative VAS pain score of 3.1 in the SILS group and 4.4 in the CLS group. Consistent with these results, Jawahar *et al.* found that patients having SILS appendectomy had considerably lower post-operative pain levels than those having traditional laparoscopic appendectomy [4]. Another meta-analysis comparing SILS and conventional laparoscopic surgery demonstrated that SILS patients experienced lower post-operative pain scores due to fewer abdominal incisions [14]. Reduced post-operative pain may facilitate early mobilization and improved patient comfort. This research found that the SILS group required less time in the hospital (2.6 versus 3.1 days), which is a decrease of around 16% in hospitalization length compared to the CLS group. This disparity was marked by statistical significance (p = 0.002). A systematic review evaluating outcomes of SILS and conventional laparoscopic colorectal surgery reported that patients undergoing SILS experienced shorter hospital stays and earlier return of bowel function compared with conventional laparoscopic surgery [5]. Ejaz *et al.* reported that single-incision laparoscopic cholecystectomy (SILC) reduces early postoperative pain and hospital stay but is associated with longer operative time and higher wound infection rates compared to conventional multi-port laparoscopic cholecystectomy [15]. Similarly, several clinical studies have reported reduced recovery time and earlier discharge in patients undergoing single-incision laparoscopic procedures due to reduced surgical trauma [8]. In the present study, post-operative complications were comparable between the two groups. Wound infection occurred in 3% of SILS patients compared with 6% in the CLS group, while port-site hernia occurred in 1% and 2% of patients, respectively. These differences were not statistically significant (p > 0.05). All things considered, the findings are in line with previous studies. According to a meta-analysis of 34 studies, conventional laparoscopy and single-incision laparoscopic colorectal surgery did not vary significantly in terms of mortality or morbidity rates [10]. Post-operative complication rates were similar between SILS and standard laparoscopic surgery, according to a meta-analysis of SILS cholecystectomy [11]. Li *et al.* reported that single-incision laparoscopic surgery (SILS) offers no significant overall clinical advantage over conventional laparoscopy, with comparable outcomes but higher intraoperative complications. However, SILS provides better cosmetic results and may be suitable in selected patients [16]. These findings indicate that SILS can be performed safely when carried out by experienced laparoscopic surgeons. Cosmetic outcome is one of the most important advantages of SILS. In the present study, 92% of patients in the SILS group reported high cosmetic satisfaction compared to 78% in the CLS group, representing approximately 14% higher satisfaction in the SILS group (p = 0.006). This result is consistent with several previous studies which have reported improved cosmetic satisfaction among patients undergoing SILS due to the single umbilical incision that hides the surgical scar [12]. Cosmetic outcomes are particularly important for younger patients and those concerned about post-operative scarring, making SILS an attractive option for minimally invasive surgery. There seems to be fewer complications, a shorter hospital stay and better cosmetic outcomes with SILS compared to conventional laparoscopic surgery, while the safety profile is comparable. However, the technique may be associated with longer operative time and greater technical difficulty, especially during the early learning phase. These findings are consistent with several randomized trials and meta-analyses comparing SILS and conventional laparoscopic surgery across different procedures [14, 16].

## Conclusion:

We show that single-incision laparoscopic surgeries are a viable and safe option. Compared to traditional laparoscopic surgery, Cosmetic results are better, post-operative discomfort is lower and patients spend less time in the hospital after SILS, while maintaining complication rates comparable to conventional laparoscopy. However, operative time is significantly longer in SILS, likely due to technical challenges and the learning curve associated with the technique. With increasing surgical experience and technological advancements, SILS has the potential to become an important approach in minimally invasive surgery.

## Figures and Tables

**Table 1:** Demographic characteristics of patients

**Parameter**	**SILS (Group A) n=100**	**CLS (Group B) n=100**	**p value**
Mean age (years)	41.6± 10.4	42.3± 11.2	0.68
Male	56 (56%)	60 (60%)	0.57
Female	44 (44%)	40 (40%)	
BMI (kg/m²)	24.8± 3.2	25.1± 3.5	0.49

**Table 2:** Comparison of operative parameters

**Parameter**	**SILS (Group A) n=100**	**CLS (Group B) n=100**	**p value**
Mean operative time (minutes)	72.4 ± 15.3	58.7 ± 13.8	<0.001
Mean hospital stay (days)	2.6 ± 0.8	3.1 ± 0.9	0.002
Intraoperative complications	6 (6%)	5 (5%)	0.76

**Table 3:** Post-operative outcomes

**Parameter**	**SILS (Group A) n=100**	**CLS (Group B) n=100**	**p value**
Mean VAS pain score (24 hours)	3.1 ± 1.0	4.4 ± 1.2	<0.001
Wound infection	3 (3%)	6 (6%)	0.31
Port-site hernia	1 (1%)	2 (2%)	0.56
High cosmetic satisfaction	92 (92%)	78 (78%)	0.006
